# Pelvic Rectal Stercoral Perforation Resulting in Diffuse Pneumatosis

**DOI:** 10.7759/cureus.9146

**Published:** 2020-07-11

**Authors:** Anupam K Gupta, Oscar A Vazquez, Miguel Lopez-Viego

**Affiliations:** 1 Minimally Invasive Surgery, University of Miami Hospital, Miami, USA; 2 Surgery, Charles E. Schmidt College of Medicine, Florida Atlantic University, Boca Raton, USA; 3 Surgery, Bethesda Hospital, Boynton Beach, USA

**Keywords:** pneumatosis, stercoral perforation, surgery, fecaloma

## Abstract

An 83-year-old woman with oral corticosteroid use for chronic autoimmune conditions presented with abdominal pain and constipation for the previous seven days. CT of the abdomen and pelvis revealed a large fecaloma with diffuse pneumatosis involving the retroperitoneum, subcutaneous tissue, and mediastinum. An emergent exploratory laparotomy revealed perforation of the rectum below the peritoneal reflection into the retroperitoneum. An end-colostomy with Hartmann's operation was then performed intra-operatively. Despite operative treatment complicated by prolonged intubation, the patient succumbed to multiorgan failure and expired.

## Introduction

A fecaloma, or stercoroma, is an impaction of dehydrated fecal matter, which results in colonic distension and increasing the pressure on the mucosal wall. As a result, the mesenteric blood supply to that region is reduced and supply ischemia sets in. The resulting ischemia due to the impacted fecal matter can lead to pressure necrosis and perforation [1-3]. Stercoral perforation due to a fecaloma is an unusual cause of perforation usually seen in elderly, debilitated, and chronically constipated patients [4]. Chronic constipation and hypomobility caused by medical conditions (hypothyroidism, cognitive impairment, diabetes, scleroderma) or medications (use of opioids, anticholinergics, antacids, and tricyclic antidepressants) may also lead to perforation [1,5]. In one study, nearly one in two patients (47%) with stercoral perforation expired from complications [6]. If perforation occurs, this may lead to peritonitis caused by fecal material and/or pus within the peritoneal cavity [7]. If the perforation occurs below the peritoneal fold, the patient may have no spillage of fecal material into the peritoneum, which may not lead to features suggestive of peritonitis.

## Case presentation

An 83-year-old woman with a prior medical history of autoimmune hepatitis, spinal stenosis, temporal arteritis, osteoporosis, and diffuse myopathy presented with abdominal pain and constipation of one-week duration. Her medications were significant for oral prednisone 40 mg daily for approximately six months for autoimmune hepatitis, laxatives, and gabapentin. The patient had a Karnofsky index of 50 (indicating she required considerable assistance and medical care). On arrival to the emergency room, the patient’s vital signs showed a heart rate of 91 beats per minute in sinus rhythm, respiratory rate of 20 breaths a minute, and oxygen saturation at 95% on room air. Her blood pressure was 129/95 mmHg. On physical examination, the patient was alert, oriented, and appeared to have abdominal bloating. There was no guarding, rebound tenderness, or rigidity. A complete blood count, metabolic profile and liver function tests were significant for a leukocytosis of 16.1x10^3^/mL, blood urea nitrogen 35 mg/dL, creatinine of 0.8 mg/dL, alanine aminotransferase 259 U/L, aspartate aminotransferase 107 U/L, alkaline phosphatase 288 U/L, total bilirubin 1.5 mg/dL, direct bilirubin of 0.4 mg/dL and PT of 11 seconds (prothrombin time/international normalized ratio [PT/INR] of 1.0). Electrolytes were within normal limits.

**Figure 1:**
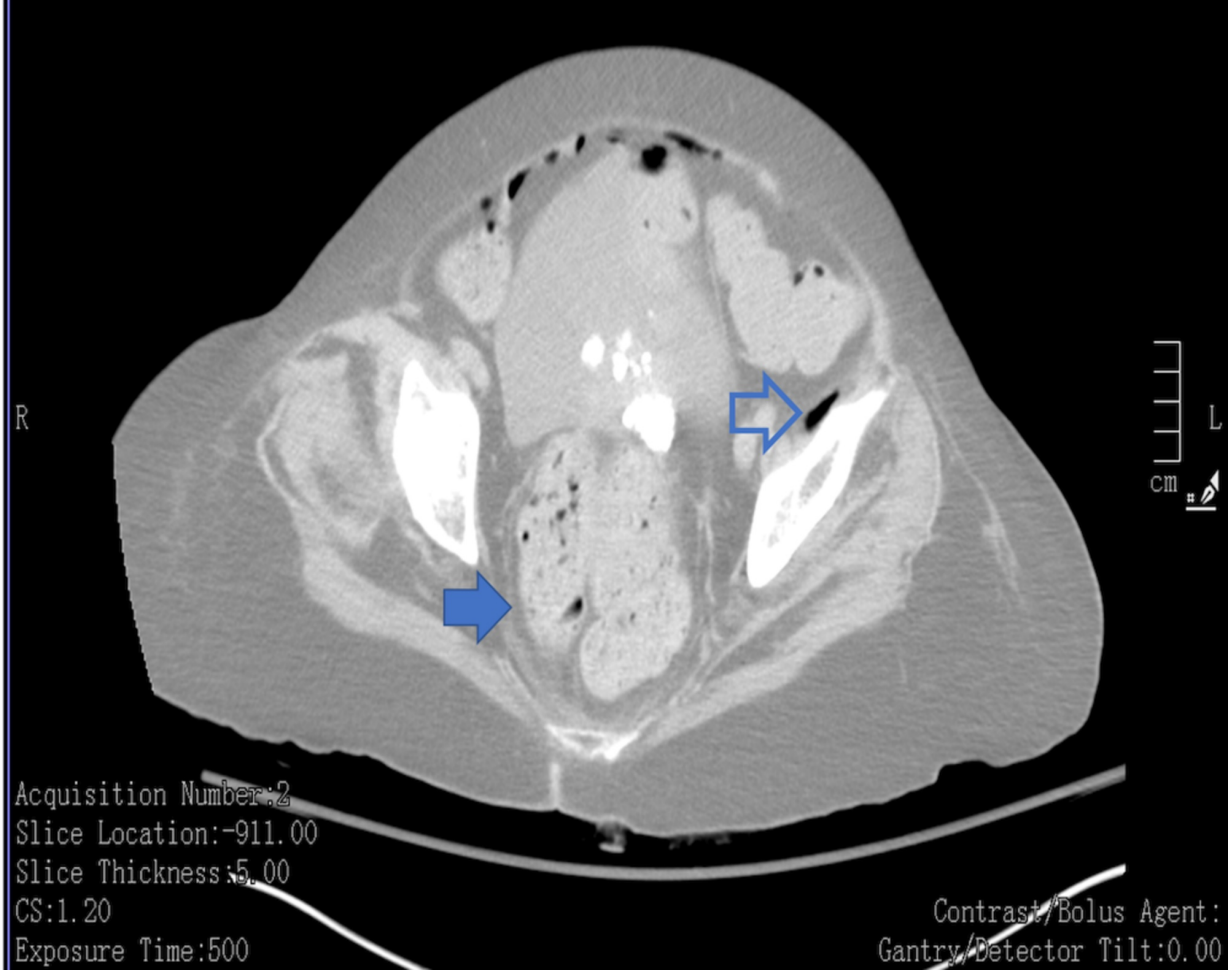
Fecaloma in rectum (solid arrow). Pneumoperitoneum with air in the pelvis (outlined arrow)

**Figure 2:**
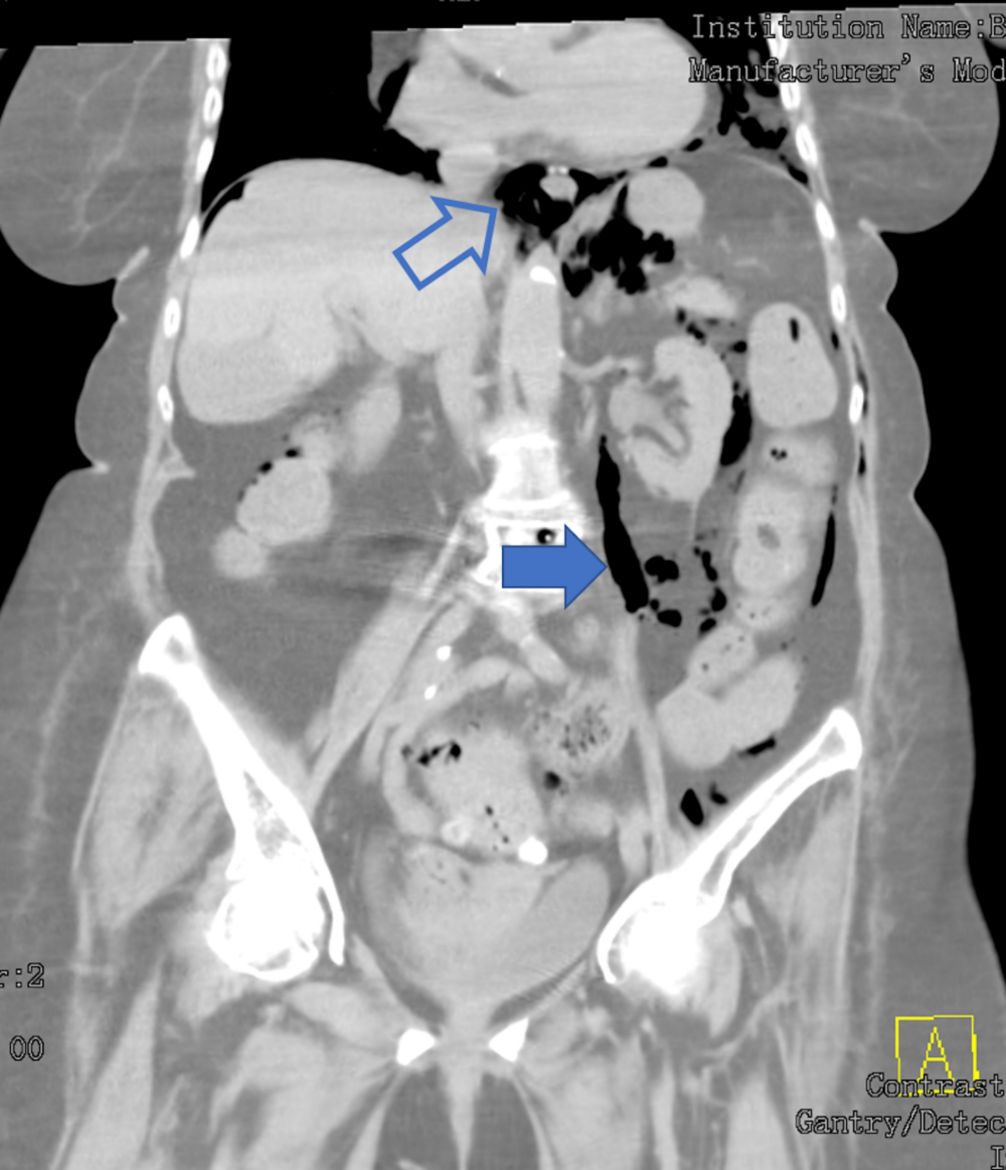
Pneumomediastinum (outlined arrow) with air in the retroperitoneum (solid arrow)

**Figure 3:**
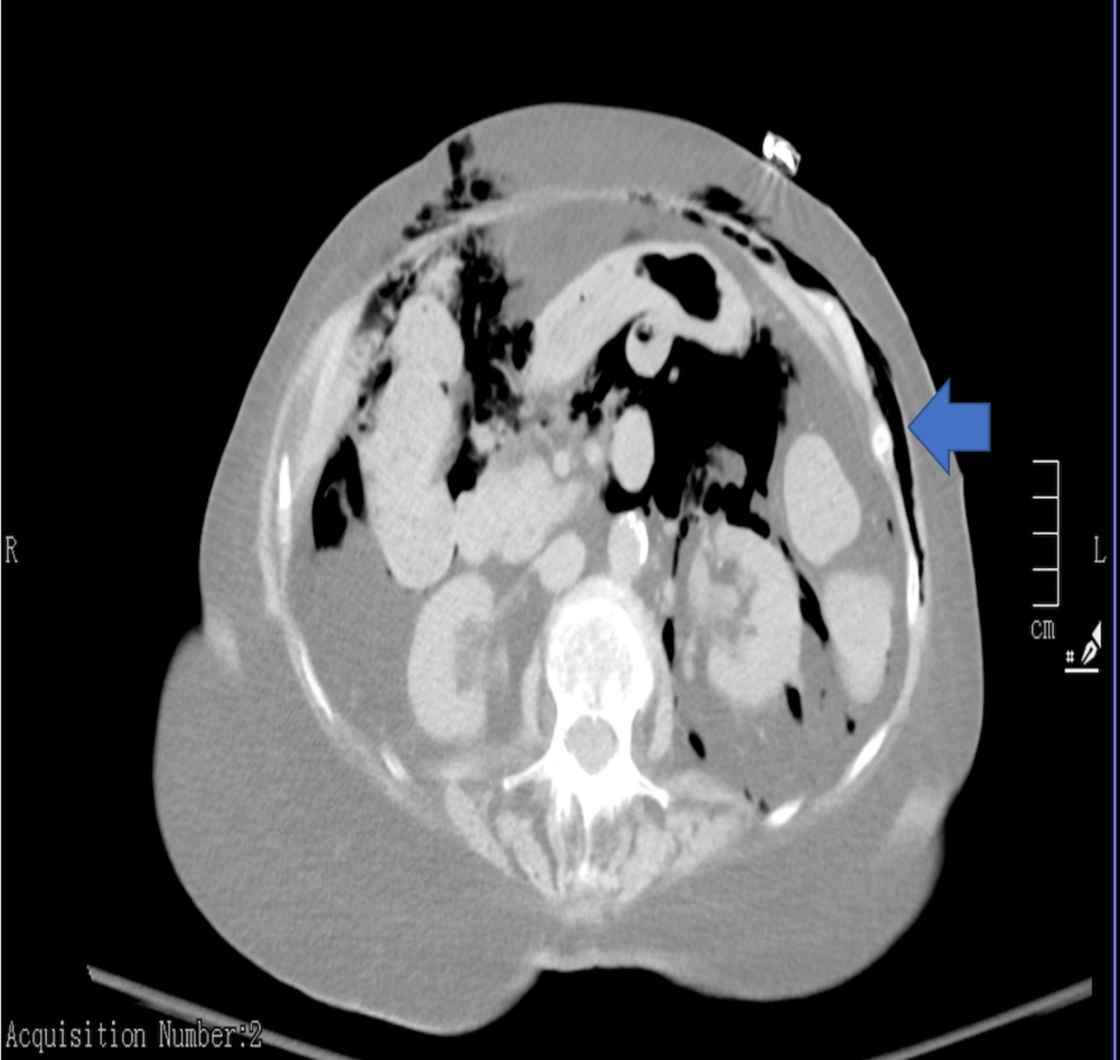
Air in the abdominal cavity, retroperitoneum, and subcutaneous tissue (solid arrow)

A non-contrast CT of the abdomen and pelvis performed in the emergency room revealed a fecaloma with pneumatosis in the mediastinum, abdomen, and subcutaneous tissue over the chest and abdomen (Figure 1, Figure 2, Figure 3). The patient was taken emergently for an exploratory laparotomy, which revealed no obvious pathology in the intraperitoneal cavity. The peritoneal reflection over the rectum and pelvic wall was opened to expose both the pelvic rectum and a cavity with fecal spillage dissecting up into the retroperitoneum. A Hartmann’s procedure with end-sigmoid colostomy was performed after thorough irrigation with sterile saline and drain placement. Pathology report of gross specimen described necrotic and inflamed tissue with perforation and no malignancy. The patient’s postoperative course was complicated with prolonged intubation and acute kidney injury, where she eventually succumbed to multiorgan failure.

## Discussion

Stercoral perforation of the colon is a rare, life-threatening entity, first reported by Berry in 1894, which represents 3.2% of all colonic perforations [1,8,9]. There is an equal incidence among males and females, and the mean age of presentation is 59 years, with an age range of 22-85 years [10]. Stercoral ulcerations are found in the intraperitoneal sigmoid colon or rectum up to 77% of the time (47% in the sigmoid and 37% in the rectosigmoid colon) potentially leading to perforation [8]. The pathophysiology involves the decreasing water content in the stool as it progresses distally in the colon and the narrow diameter and high intraluminal pressures in the distal colon [9]. The three most common locations for stercoral ulceration are the anterior rectum, just proximal to the peritoneal reflection, the antimesenteric border of the rectosigmoid junction, and the apex of the sigmoid colon. Most commonly seen on the antimesenteric side of the bowel wall. These locations have relative ischemia as blood supply to the bowel enters on the mesenteric side [7,11].

Stercoral perforation commonly occurs in bedridden patients with a history of chronic constipation and may present with abdominal pain, rectal discomfort, fecal incontinence, anorexia, nausea, vomiting, paradoxical diarrhea, urinary frequency, melena, and urinary overflow incontinence [12]. Most patients have an elevated white blood cell count with a left shift, but physical examination or laboratory tests are not always reliable for diagnosing stercoral colitis [13]. In patients with fecal impaction, CT scans may reveal the presence of stercoral colitis. Chest x-rays may only reveal free air under the diaphragm in 30% of cases of colonic perforation [14]. In uncomplicated fecal impaction, there is colonic distension, and the wall is thin; however, in stercoral ulceration, focal thickening of the colonic wall may be present caused by edema from the ischemia and ulceration. Stranding of the pericolonic fat in a segment that shows fecal impaction suggests colonic edema or ischemia, and the presence of extraluminal bubbles of gas, or an abscess, suggests that perforation has already occurred [7]. CT also has a reported accuracy ranging from 82% to 90% [15]. Histopathology findings are usually significant for ischemic necrosis and nonspecific inflammatory changes [16].

Suggested criteria by Maurer et al. for diagnosing stercoral perforation include (a) round or ovoid perforation of more than 1 cm in diameter on the colonic antimesenteric side, (b) the presence of fecaloma within the colon, extending through the perforation site, or spillage within the peritoneal cavity, (c) chronic inflammatory changes along with pressure necrosis and ulcer formation on microscopic examination, and (d) external injury, diverticulitis, and/or lack of obstruction due to cohesion or tumor [17]. Surgical management of stercoral perforation includes open laparotomy, massive peritoneal lavage, and Hartmann's procedure with a sigmoid colostomy or segmental resection with primary anastomosis and diverting colostomy [16,18]. Intraoperatively, colon disimpaction, colonoscopy, identification of additional stercoral ulcers, and removal of affected colon segments may prevent secondary perforations and further complications. Treatment of intra-abdominal sepsis can be achieved by perforation control and institution of broad-spectrum intravenous antibiotics against common gram-negative and anaerobic pathogens cultured from the peritoneal fluid (e.g., E. coli, E. faecalis, and B. fragilis) [16,19]. It is rare to see stercoral perforation in the pelvic rectum below the peritoneal fold and no signs of peritonitis due to lack of intra-abdominal spillage, as seen in the patient presented, along with diffuse pneumatosis extending into the retroperitoneum, subcutaneous tissue, and mediastinum. This unusual location is possibly due to her debilitated status and chronic steroid use. Finally, treatment remains surgical with source control and management of comorbidities.

## Conclusions

Stercoral perforation of the rectum below the peritoneal reflection is an unusual entity that may be challenging to diagnose as a patient may not present with peritonitis and radiological findings may not be conclusive because of diffuse pneumatosis. Hartmann’s procedure with end-colostomy and source control should remain the primary management.

## References

[1] Kanwal D, Attia KME, Fam MNA, Khalil SMF, Alblooshi AM (2017). Stercoral perforation of the rectum with faecal peritonitis and pneumatosis coli: a case report. J Radiol Case Rep.

[2] Grinvalsky HT, Bowerman CI (1959). Stercoraceous ulcers of the colon: relatively neglected medical and surgical problem. JAMA.

[3] Lal S, Brown GN. (1967). Some unusual complications of fecal impaction. Am J Proctol.

[4] Celayir MF, Köksal HM, Uludag M (2017). Stercoral perforation of the rectosigmoid colon due to chronic constipation: a case report. Int J Surg Case Rep.

[5] Tessier DJ, Harris E, Collins J, Johnson DJ (2002). Stercoral perforation of the colon in a heroin addict. Int J Colorectal Dis.

[6] Brombacher GD, Murray WR (1998). Emergency subtotal colectomy for chronic constipation. Scott Med J.

[7] Heffernan C, Pachter HL, Megibow AJ, Macari M (2005). Stercoral colitis leading to fatal peritonitis: CT findings. Am J Roentgenol.

[8] Berry J (1894). Dilatation and rupture of sigmoid flexure. Br Med J.

[9] Serpell JW, Nicholls RJ (1990). Stercoral perforation of the colon. Br J Surg.

[10] Sharma M, Agrawal A (2010). Case report: stercoral sigmoid colonic perforation with faecal peritonitis. Indian J Radiol Imaging.

[11] Tokunaga Y, Hata K, Nishitai R, Kaganoi J, Nanbu H, Ohsumi K (1998). Spontaneous perforation of the rectum with possible stercoral etiology: report of a case and review of the literature. Surg Today.

[12] Kumar P, Pearce O, Higginson A (2011). Imaging manifestations of faecal impaction and stercoral perforation. Clin Radiol.

[13] Saksonov M, Bachar GN, Morgenstern S, Zeina AR, Vasserman M, Protnoy O, Benjaminov O (2014). Stercoral colitis: a lethal disease-computed tomographic findings and clinical characteristic. J Comput Assist Tomogr.

[14] Kwag SJ, Choi SK, Park JH, Jung EJ, Jung CY, Jung SH, Ju YT (2013). A stercoral perforation of the rectum. Ann Coloproctol.

[15] Kim SH, Shin SS, Jeong YY, Heo SH, Kim JW, Kang HK (2009). Gastrointestinal tract perforation: MDCT findings according to the perforation sites. Korean J Radiol.

[16] Bhatt VR, Murukutla S, Dipoce J (2014). Perforation in a patient with stercoral colitis and diverticulosis: who did it?. J Community Hosp Internal Med Perspect.

[17] Maurer CA, Renzulli P, Mazzucchelli L, Egger B, Seiler CA, Büchler MW (2000). Use of accurate diagnostic criteria may increase incidence of stercoral perforation of the colon. Dis Colon Rectum.

[18] Guyton DP, Evans D, Schreiber H (1985). Stercoral perforation of the colon. Concepts of operative management. Am Surg.

[19] Huang WS, Wang CS, Hsieh CC, Lin PY, Chin CC, Wang JY (2006). Management of patients with stercoral perforation of the sigmoid colon: report of five cases. World J Gastroenterol.

